# Nanoscale Lithium Quantification in Li_X_Ni_y_Co_w_Mn_Z_O_2_ as Cathode for Rechargeable Batteries

**DOI:** 10.1038/s41598-018-33608-3

**Published:** 2018-12-04

**Authors:** Stéphanie Bessette, Andrea Paolella, Chisu Kim, Wen Zhu, Pierre Hovington, Raynald Gauvin, Karim Zaghib

**Affiliations:** 1 Hydro-Québec, Centre d’excellence en électrification des transports et stockage d`énergie, Varennes, J0L 1N0 Canada; 2 McGill University, Department of Mining and Materials Engineering, Montréal, H3A 0C5 Canada; 3 Consulting Hovington, Boucherville, Québec, Canada

## Abstract

Time-of-flight secondary ion mass spectrometry (TOF-SIMS) using a focused ion-beam scanning electron microscope (FIB-SEM) is a promising and economical technique for lithium detection and quantification in battery materials because it overcomes the limitations with detecting low Li content by energy dispersive spectroscopy (EDS). In this work, an experimental calibration curve was produced, which to our best knowledge allowed for the first time, the quantification of lithium in standard nickel manganese cobalt oxide (NMC-532) cathodes using 20 nm resolution. The technique overcomes matrix effects and edges effects that makes quantification complex. This work shows the high potential of TOF-SIMS tool for analytical characterization of battery materials, and demonstrates its tremendous capabilities towards identification of various chemical or electrochemical phenomena in the cathodes via high-resolution ion distributions. Various phenomena in the ion distributions are also assessed, such as edge effects or measurement artifacts from real signal variations.

## Introduction

Scanning electron microscopes (SEM) are essential tools for microstructure characterization and microanalysis of new materials, and their ease of use has made them one of the most widespread tools for analysis of structures down to the nanometer scale^1^. Furthermore, the characteristic x-rays from the beam-specimen interactions allow chemical identification of the samples, when observed by an energy-dispersive x-ray spectrometer (EDS) detectors in the microscope chamber. In battery R&D, conventional EDSs are not very useful for detection of lithium (the key element in a battery) because the characteristic x-rays (Li K ~ 55 eV) have too low energy, and are absorbed in the instrument window, typically made of beryllium or polymer. Special windowless EDS detectors with optimized electronics^2^ were developed to permit lithium detection and have proven effective to detect lithium soft x-rays in pure Li-metal and binary compounds^2,3^. However, it has been demonstrated that even an optimized windowless EDS has limitations for Li detection and quantification due to its low sensitivity (>20 wt%) and lack of understanding of Li x-ray emission processes^3^. In addition, the mass absorption coefficient (MAC) of Li K x-ray within the matter is high, adding to the difficulty to detect lithium. Finally, lithium is a light element with a low fluorescence yield, of the order of 10^−4^ ^4^, which means that electron beam excitation of the lithium core shells results in prevailing relaxation into Auger electrons, therefore the lithium x-ray signal in SEM is very low.

Other alternatives to EDS, which include Electron Energy-Loss Spectroscopy (EELS) and Secondary ion mass spectrometry (SIMS), have limitations for lithium detection. EELS collects the energy distribution of electrons after their passage through a specimen, giving information about the chemical composition of the sample (ionization edges) and bonding states through edge fine structures. Mounted on a TEM or STEM, an EELS detector is promising for lithium detection in materials since it can detect all energy-loss events, therefore low fluorescence yield of lithium is not a constraint as in EDS. However, electron beam sensitivity is a concern as with EDS analysis. In addition, EELS measurements require adequate thin foil sample preparation, otherwise the lithium K edges can be masked by plasmon peaks^5^. Another drawback of EELS for analysis of battery materials is that the M_2,3_ edges of the transition metals used in typical cathode compounds (Mn, Fe, Ni and Co) are very close to the Li K edge, therefore some information about the lithium fine structure can be shadowed by the transition metals^6^. It is possible to indirectly analyze lithium in these compounds by correlating the effect of lithium bonding on the change of transition metals and oxygen chemical states and fine structures. SIMS appears to be an adequate solution to the detection limits of EDS, since being a physical sputtering technique, it relies on collision cascades created by energetic ions rather than electronic transitions following excitation of an atom by a primary beam electron. It allows high surface sensitivity, as well as full coverage of the elements in the periodic table, including lithium. Typically dedicated SIMS instruments (TOF-SIMS or magnetic sector analyzers) allow 50 nm resolution along with high mass resolution (>3000) and high sensitivity (ppb) under ultra high vacuum conditions (10^−9^ Torr)^7–9^. A compact C-TOF SIMS detector by TOFWERKS is available on the market that offers 4 ppm mass accuracy together with sufficient mass resolution (800) to successfully distinguish lithium, transition metals and isotopes. Combining a C-TOF detector in a focused ion-beam scanning electron microscope (FIB-SEM) will allow unique imaging and analysis capabilities of bulk battery samples. In addition, the FIB probe used as ion source allows a higher spatial resolution (40 nm recently reported with Ga FIB^10^) than some dedicated SIMS instruments. However, Ga FIB permits commonly 10 nm probe sizes^11^, TOF-SIMS mounted on a FIB-SEM platform is therefore a ‘*pragmatic*, *cost-effective’*, high-resolution technique in comparison to EELS and dedicated SIMS instruments, although it may not reach the latter’s sensitivity (ppb)^12,13^.

SIMS has been used so far in battery R&D mostly as a qualitative tool. It provides information on the distribution of active materials in LiCoO_2_ cathodes^14^, on the effect of polymer additives on lithium-ion distributions in lithium iron phosphate (LFP) cathodes^15,16^, permitted study of solid electrolyte interphase (SEI) formation during charge-discharge cycles^17,18^ and quality control of manufactured electrodes. Furthermore, it is also an important tool to identify lithium-ion hotspots trapped in grain boundaries as a result of battery cycling^19^. To the best of our knowledge, the quantification of Li in battery materials and studies of edge effects and matrix effects using TOF-SIMS mounted on a Ga FIB microscope has not been successfully performed.

The edge effects are observed as local variations in signal intensity arising from the change in sputtering yield caused by topography of the sample. The sputtering yield, which is proportional to the intensity in the SIMS spectrum, is an increasing function of the angle of incidence (θ). It has a dependency of 1/cos(θ) from 0 to ~70°^20^, where it is demonstrated experimentally that the function reaches a maximum and falls to 0 at 90°^21^. Therefore, edge effects can easily lead to misinterpretation of the content of an element in a sample by showing regions with higher intensities that are artifacts related to the increased sputtering. In semiconductor materials, these edges effects are mainly seen at the edges of the crater formed during SIMS analysis. A simple electronic gating [filter] of the data collected can be used to remove the crater edge effects by sputtering a larger area than the acquisition area^22^. Considering that (a) battery materials are inhomogeneous and (b) the cycling process creates defects in the particles (cracks, pores, etc…), identifying the edge effect is made more complex and this gating technique cannot be applied.

Matrix effects are variations of the yield of secondary ion species in a chemical environment where an element of interest is found. A quantification scheme exists for SIMS analysis, which implies correction of the matrix effects, but is developed and proven for trace elemental analysis, mostly of dopants in homogeneous and simple matrices such as silicon. This scheme needs an implanted secondary standards of known composition to reproduce emission conditions and from which matrix correction factors, so called relative sensitivity factors (or RSF) are computed and used to correct the intensity collected in the SIMS measurement^23^. RSFs are specific to the element of interest, the matrix and the ion bombardment under which the analysis is done. Quantification of matrix levels (by definition more than 1 percent atomic), can be achieved using SIMS, requiring implanted standards of the species of interest^23,24^. It has been achieved, to our best knowledge, only with semiconductor-type materials by the study of depth profiles. Battery materials have complex stoichiometry and inhomogeneous structures: it means that a quantitative model must be developed for SIMS since identification of lithium content is of primary importance in this field.

In this work, a Time-of-Flight SIMS (TOF-SIMS) detector (that allows rapid and parallel detection of all elements) was used to quantify, for the first time, Li in standard nickel manganese cobalt oxide (NMC-532) cathodes. It will be shown that the matrix effects can be assessed by an experimental calibration curve and that edge effects can be understood and distinguished from other phenomenon in ion distributions. The aim of this work is to develop a quantitative technique to be used as a complement to EDS for lithium ions in battery materials.

## Results and Discussion

### Matrix effects in NMC

**Figure 1:**
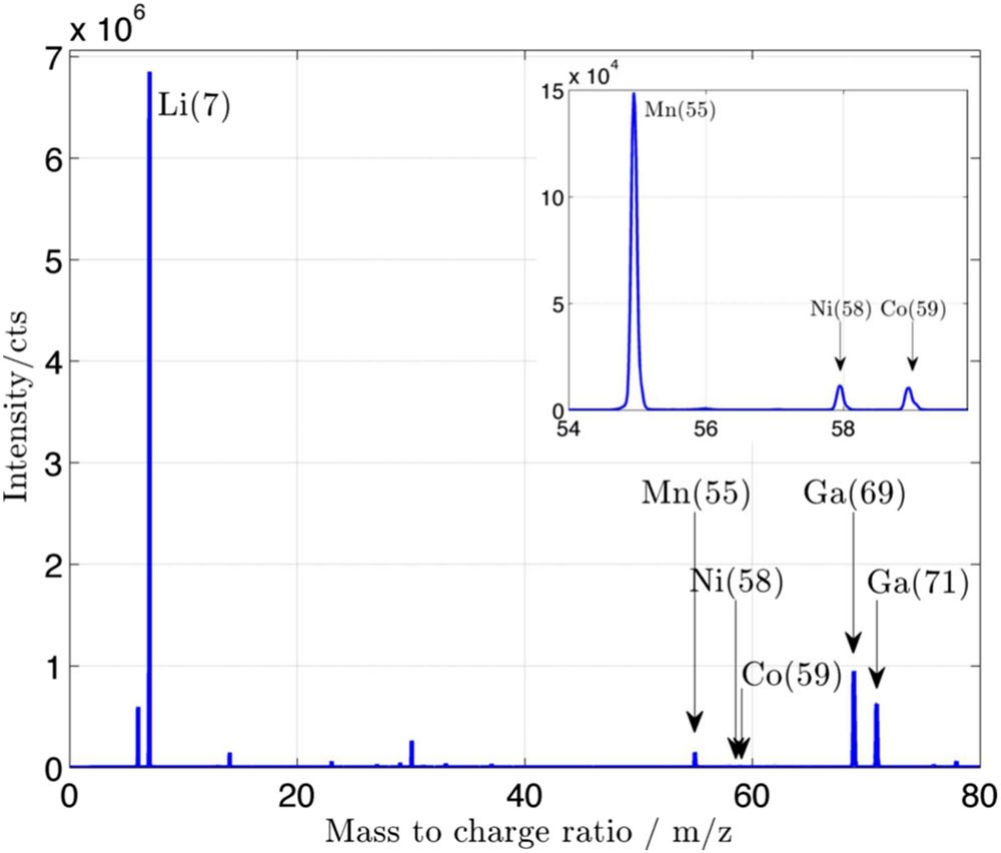
(**a**) Mass spectra of Li in NMC cathode with full lithium intercalation. Identification of Li(7), Mn(55), Ni (58), Co(59) as well as Ga (69) and (71). (**b**) Expanded view of m/z region from 54 to 60.

Figure 1 shows a typical SIMS spectrum obtained in positive mode at a representative location on a fully lithiated NMC-532 cathode. A strong lithium peak and the presence of nickel, cobalt and manganese are observed, as well as gallium isotopes from the primary ion source. Lithium compounds with transition metals or/and with oxygen in small quantities are also found in the mass spectra (3 orders of magnitude smaller than Li peak) but not identified on the figure. Ni, Co and Mn show smaller intensities compared to lithium, as seen in the expanded view in Fig. 1. These smaller intensities are explained by lithium being in SIMS is one of the most sensitive elements since its low mass and low binding energy gives a high sputtering yield^25^. Matrix effects can further emphasize the emission of Li atoms from the NMC compound. In this next section, we will prove that matrix effects in the samples prevent us from direct quantification of lithium in the compound. According to theory, chemical bonding in a material can either prevent or enhance atom extraction compared to the signal received from a pure sample of the element of interest^23^. Comparing the SIMS signal with pure lithium metal and the Li signal from NMC in this case proves the existence of matrix effects that emphasize lithium extraction. Under similar bombardment conditions and ion dose, our experiments show that Li intensity is 1.76 times higher in the compound that in the pure material. Using the Transport of Ion in Matter (TRIM) program (part of the Stopping and Range of Ion in Matter (SRIM) software)^26^ which allows calculation of the sputtering yield of elements in a variety of compounds, we can study the theoretical behavior of lithium emission. From the results in Table 1, we calculate the theoretical emission ratio of lithium in the two matrices and compare it to the ratio of intensities obtained experimentally where the intensity collected in SIMS is directly proportional to the sputtering yield. TRIM renders a theoretical ratio of lithium in the NMC matrix versus in pure Li matrix of 0.76. The theoretical ratio doesn’t take into account preferential sputtering, which can cause stoichiometry change in the compound. It is possible that enhancement of sputtering yields in the experimental results is due to surface roughening of the sample over the analysis time^27^. The software computes the surface sputtering analysis using a flat, always intact amorphous surface. In our measurement, we took into account regions of interests (ROI) (regions of 10 × 10 pixels or less) that were free from edge effects, and we stipulate that locally the surface is homogenous. This way, we conclude that matrix effects are occurring in NMC and affect the emitted signal. From Table 1, it is also observed that the sputtering yield of elements in NMC is inversely proportional to their surface binding energy^20^. This fact, and considering that lithium is a light atom in a matrix composed of heavier elements, can contribute to cause the particularly strong sputtering of Li in the NMC compound and adds to the influence of chemistry of the material on the lithium signal intensity.

**Table 1:** Sputtering yield of Li in pure lithium and Li Mn, Ni, Co in cathode material calculated by TRIM software^26^ (using default 99999 ions in surface sputtering) from Ga^+^ bombardment at 30 keV.

Element	Matrix	Sputter yield (Y) (u.)	Surface binding energy (SBE) (eV)
Li	Li	2.2500	1.67
Li	NMC	1.6700	1.67
Ni	NMC	0.4849	4.46
Co	NMC	0.1919	4.43
Mn	NMC	0.3964	2.98

**Figure 2:**
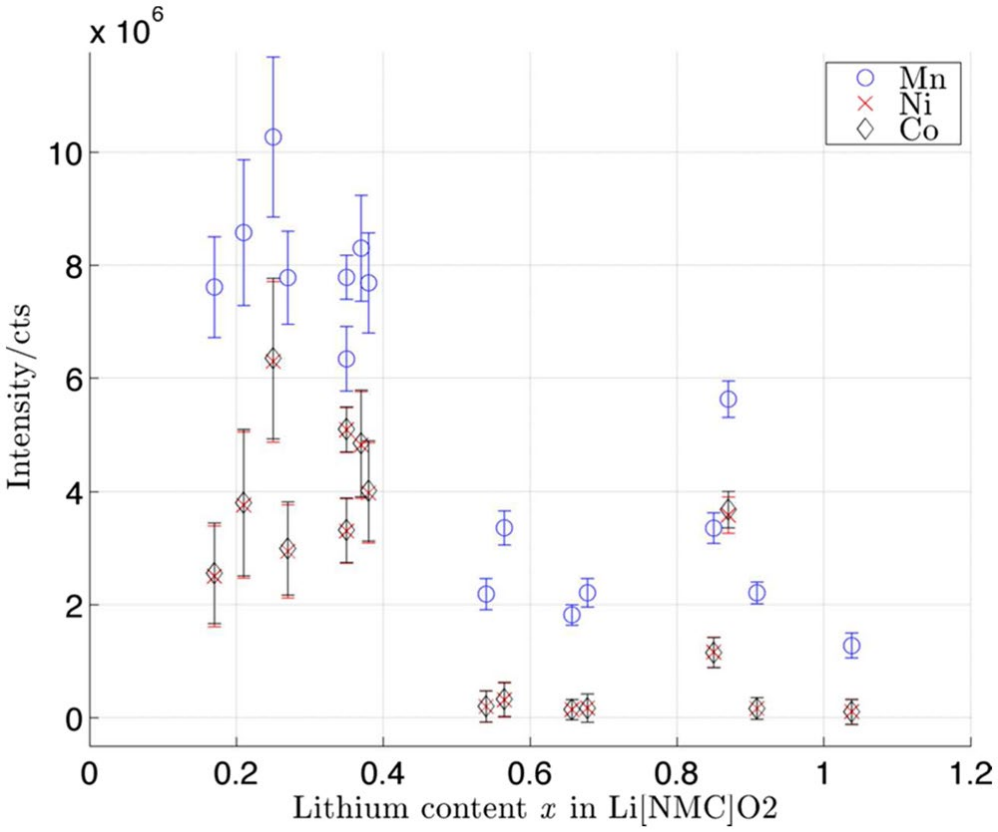
Variations of the intensities of the active elements in NMC compound (Mn, Ni, Co) versus the lithium stoichiometry (*x* of Li) in cathodes electrochemically cycled and at different state of charge.

It is clear that Li suffers from matrix effects in NMC compound, and steps must be taken to correct the collected intensities to properly quantify the lithium content in the NMC cathode. As mentioned before, the existing quantification method suggests the determination of RSFs. Computation of these factors imply that the concentration in the reference element and the standard^23^ is constant in the sample Usually, the reference element is present in significant concentrations in the specimen sample. This major component happens to be, for example the Si or Ge matrix, and the elements of interest are present only in trace amounts, therefore the reference intensity remains constant. In this case, since lithium is the major constituent and element of interest, another reference must be chosen. The reference should be selected from the other active elements in NMC. However, Fig. 2 shows the intensity of Mn, Ni and Co in the cathode at different state of charges (lithium content *x* in the material). For all of the elements, a net decrease in the intensities was observed as well as a poor statistical correlation. The first phenomena is explained by the fact that intercalation of lithium in the compound affects its chemistry, so Mn, Ni and Co suffer a decrease in mass fraction in the compound as lithium stoichiometry passes from 0 to 1. The absence of a linear correlation in the curves is explained by few collected counts of Mn, Ni, Co, resulting from low sputtering yield as seen in Table 1. As shown in Fig. 1, it is possible to observe that the lithium peak is an order of magnitude higher than the manganese peak in the compound. In Fig. 2, we see that manganese has intensity 10 times higher than both Ni and Co, due to its lower surface binding energy. Ni and Co are neighboring elements in the periodic table, which contributes significantly to the mass effect, with the lighter atoms being ejected preferentially from the surface. Therefore a small variation in the shape of the Mn, Ni, Co peaks on the mass spectrum can lead to a noticeable difference in the integrated intensity. Since the intensity and the concentration of Mn, Ni and Co fluctuate in the compounds, it is not possible to consider them as invariable references in order to determine the concentration of lithium. Finally, another argument that plays against the use of RSFs with our cathodes is that the computation of the RSF correction factor also implies the estimation of the depth of the sputtered crater on the analysis region as a result of destructive sputtering. Taking Si in implanted semiconductor samples as an example, sputtering on this monocrystalline material creates a square uniform crater, and its depth is assessed by SEM imaging of stylus profilometry in the equipment^23,28^. However, NMC particles are mainly constituted of agglomerated primary particles of ~500 nm, and therefore the samples exhibit different and random crystallographic orientations with respect to the primary ion beam. Adjacent grains with different crystallographic orientation suffer from different milling rates^29^, making the determination of the crater depth impossible since the sputtering technique reveals grains lying underneath the surface ones, which might be orientated differently.

### Calibration curve of Li in NMC

Since the mass spectra is mostly composed of lithium counts and because of failed attempts to compute RSF with the inhomogeneous and complex stoichiometry of NMC, construction of an experimental calibration curve based solely on the experimental lithium intensity was the best option to comply with the nature of battery materials.

**Figure 3:**
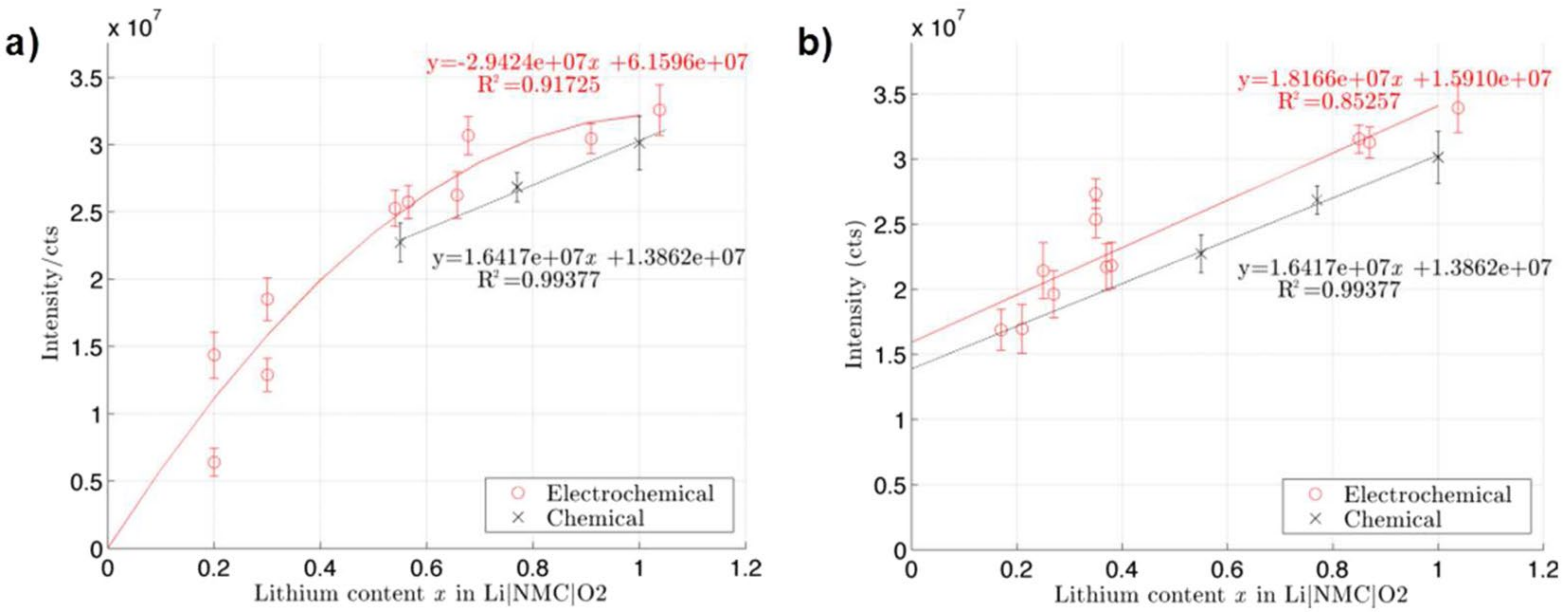
Calibration curve of lithium in Li_x_NMCO_2_ (**a**) C/10 charge rate and (**b**) C/50 charge rate.

Figure 3 represents the electrochemical calibration curves under different cycling rates. The y-axis is the measured lithium intensity (in counts) while the x axis is the lithium content (or stoichiometry) in the NMC compound. The amount of lithium (*x*) in the cathode is inversely proportional to the state-of-charge of the battery; therefore a fully discharged battery corresponds to a cathode with full lithium intercalation (*x* = 1). To obtain this curve, it should be noted that measurements of the lithium intensity for small regions of interest (ROI) at the surface of grains are not affected by edge effects. The next section will explain how we have distinguished them. For each value on the curve in Fig. 3, an extensive number of acquisitions of 15 × 15 μm regions were taken on each of the NMC samples, and multiple ROIs defined within the analysis areas in order to obtain good statistics. All analyzed NMC particles were larger than the acquisition area and the particle edges were not included in the analysis. X-ray diffraction analysis of the cycled cathodes was done to confirm the lithium content in the materials (Supplementary Figs S1 and S2). Similarly to RSFs, this calibration curve allows the quantification of Li in NMC, with gallium bombardment at 30 keV and 500 pA (analysis conditions). Corrections must be applied for different analysis conditions (different view field, and current) to account for the change in applied dose over the analysis area (higher dose triggers higher sputtering). Therefore, this curve is specific to NMC and to gallium bombardment. Hence, if lithium concentration is to be determined in another battery sample or with another ion source, a new calibration curve must be constructed.

Both electrochemical and chemical delithiation processes have been investigated to assess bulk phase distribution of polycrystalline cathode materials and to help understand the role of the electrolyte on the material^30^. In this work, standard NMC powder was chemically delithiated using dibromine as an oxidant, then analyzed with TOF-SIMS under the same conditions as used previously. Three samples were made by using different amounts of the oxidizing agent (ranging from none to in excess) to produce NMC powders with different degrees of lithiation, which are obtained chemically. The powders were then dried and prepared for observation under the microscope without cycling in a battery. The dashed curve in Fig. 3 relates to these results. Note that since Br_2_ is a mild oxidant (with redox couple 4.01 V against Li^+^/Li), it cannot delithiate NMC compound further than x = 0.5. The chemical delithiated curve for lower lithium content (*x*) was extrapolated. Both curves are fairly parallel, the difference ΔI represents the added analytical signal of lithium that can be associated to residual lithium from the electrolyte salts.

Figure 3a shows the lithium intensities in TOF-SIMS for cathodes cycled at C/10. The intensities show a highly polynomial behavior that can be extrapolated towards the origin. This polynomial behaviour is confirmed by theoretical TRIM simulations of the lithium sputtering yield in NMC compounds (Supplementary Fig. 3). The experimental curves show rapidly decreasing intensities at low lithium content (x < 0.5). Wu *et al*. showed that the lithium diffusion coefficient increases with increasing SOC - that is Li diffusion is facilitated in compounds with low lithium stoichiometry^31^. This higher diffusion coefficient at low Li content could explain the observed sharp decrease in intensity below x = 0.5. XRD measurements on the cathodes with low lithium content (x ≈ 0.2 and 0.3) showed distinct phase heterogeneities such that indexing the Li content was impossible, explaining the variability in the recorded intensities in the graph. Many studies have noticed state-of-charge (SOC) heterogeneities, which lead to Li-rich and Li-poor phases in the secondary particles. These heterogeneities are usually enhanced by high cycling rates, and can lead to capacity fade and local overcharge or discharge^30^. In an attempt to reduce the phase inhomogeneity, new coin-cells were prepared and cycled at very low rates, the lower current favouring more stability in lithium intercalation in the compound, as confirmed by XRD (see Supplementary Fig. S1).

Figure 3b reports the results of the NMC cathodes cycled at C/50. The electrochemical calibration curve at C/50 exhibits an extrapolated non-null y-intercept, ΔY, as does the chemical curve in which we expect null lithium intensity if the cathode was completely delithiated. Especially at low lithium content, a C/50 charging rate resulted in overestimation of the lithium content in the material. Local segregation of lithium and deactivated particles may explain the added lithium signal. Cracking of primary particles as a result of cycling can further emphasize SOC heterogeneities^30^ as well as relative position of the secondary particles within the cathode itself, where gradients of lithium ion concentrations were observed along the thickness of the NMC cathodes and in function of the particle size^32^. XRD studies have also shown that the crystalline NMC matrix suffers from anisotropic volume change during both chemical and electrochemical delithiation, leading to stress within the matrix. The primary particles may then be subject to disconnection from the NMC matrix, leading to the observed bulk inhomogeneities since the primary particles no longer participate in the delithiation process^30,33^. Isolated particles from the electrochemical network retain more lithium compared to the connected matrix^32^, and might explain the added ΔY in the calibration curve. We think the residual lithium seen in the TOF-SIMS intensities is not a consequence of sample preparation or handling, but rather a quantity of lithium ions that is not possible to extract from the material – whether chemically or electrochemically. Finally, another avenue to explain ΔY is the formation of a surface reconstruction layer in the secondary particles, creating a gradient layer on the surface of the particles, driven by the high reactivity of oxygen close to the particle surface^30^. During both delithiation processes, Ni becomes somewhat oxidized and Li depleted at the surface. Tian *et al*.^30^ found that chemical delithiation with strong oxidizing agents lead to different surface chemistry than electrochemical processes, an effect that was observed for the highest SOC (low lithium content) with NO_2_BF_4_. This disparity could explain the variability between the extrapolated two curves in ΔY. This phenomenon was also observed at the nanoscale in LiNMO (where N = Ni, M = Mn) batteries and has proven to lead to increase of transition metal concentration as well as shortage of lithium at the surface of the particles^33^. By using very low changing rates, the SOC inhomogeneities have a larger time to set, making it more difficult to extract Li ions during discharge.

### Assessment of edge effects in ion distributions and other phenomena

**Figure 4:**
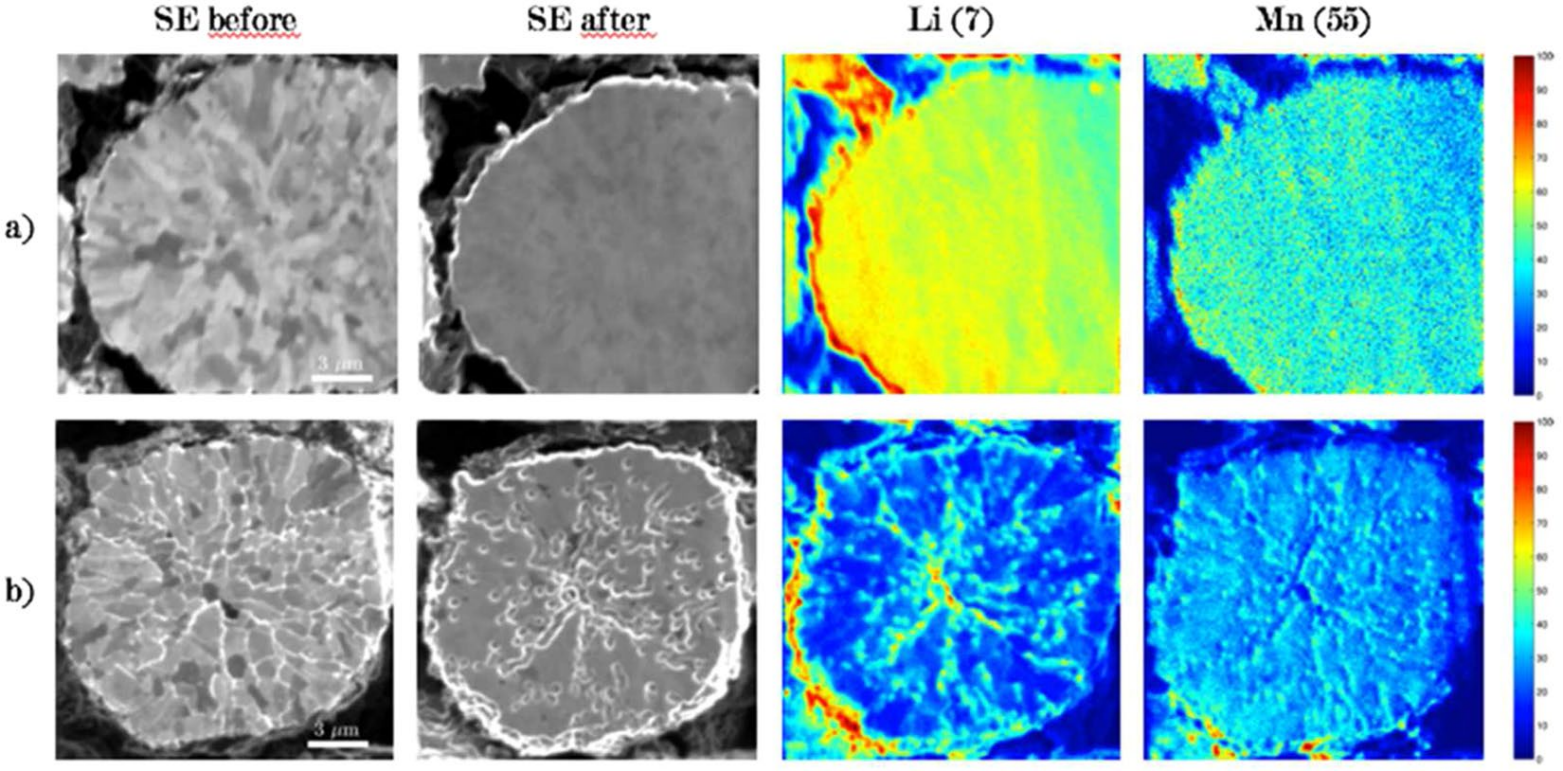
FIB SE images before and after analysis and ion distributions of Li, Mn of pristine (**a**) and cycled (**b**) cathode. The ions maps represent the cumulative data over 100 frames of analysis and normalized according to the maximum intensity in the species for each sample.

Figure 4 shows SIMS ions distribution of the active elements in the NMC cathodes, as well as before and after FIB secondary electron (SE) images of the analysis regions for a) a pristine sample (uncycled) and b) an electrode with SOC = 100%. The image before and after SIMS analysis allows us to fully understand the effect of the analysis on the samples, as well as the effect of cycling. Before the analysis, the FIB SE image reveals NMC secondary grains of spheroidal shape, containing primary grains with clear contrast variations. The SE images after analysis for both samples show loss of contrast in the primary grains, associated with Ga ion implantation in the material which causes amorphization^25^, as well as modification of the grain surface. Furthermore, sputtering is a destructive technique which removes material layer by layer from the material surface. On the cycled sample, it is evident that cycling changed the morphology of the NMC grains, creating cracks in the particles at grain boundaries. These cracks, which allow a change in topography of the samples, are emphasized during the cycling and allow enhanced sputtering since these positions have a favorable angle of incidence with the primary ion beam. These cracks lead to higher intensity of ion distributions and can be identified as edge effects. Edge effects are also expected at the periphery of particles, which are also observed in samples a and b. Differences in milling rates between adjacent grains of different crystallographic orientation^29^ can also emphasize creation of edge effects, since grains in a channeling direction will have lower milling rates because the collision cascades occur deeper in the material^34^, and hence sputtering is reduced.

Therefore, to determine the true concentration of lithium in the samples, edge effects must be distinguished from real signal variations from local compositional changes, and to do so, another source of information is needed to properly interpret the data. According to the FIB SE images in Fig. 4, it is possible to link regions with altered topography that present high brightness on the SE image and with the occurrence of edge effects in ion distributions. Since high brightness indicates generally high topography, these regions can be disregarded for determining the Li intensity of the NMC grain. Confocal microscopy can also be used for surface analysis (after sputtering) to view the topography. Work is currently underway, but not shown here, to link roughness from confocal measurements to intensities of the elements in the ion distributions.

**Figure 5:**
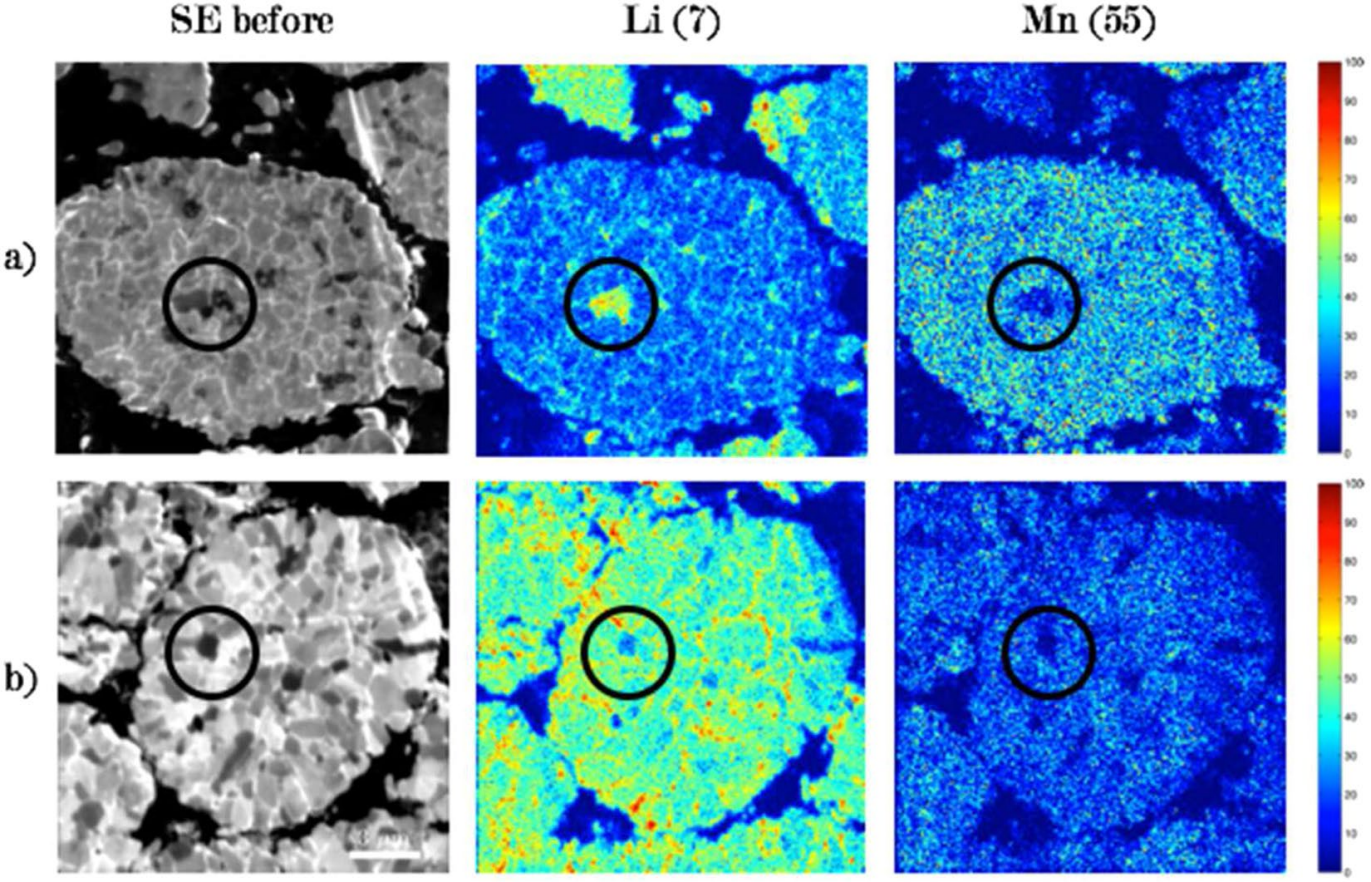
FIB SE image before TOF-SIMS analysis and ion distributions (Li and Mn) showing identification of (**a**) crystallographic effect and (**b**) electrochemical effect related to the apparent intensity of lithium in channeling grain versus the background matrix (of non-channeling orientation). Ion map distributions were normalized to the maximum in each species for each sample. In both cases, manganese follows the expected trend of a channeling direction.

Not all regions with high intensities are related to edge effects. It is possible to observe grains with high lithium counts associated with a channeling crystallographic direction (low brightness) on the FIB SE image as shown in Fig. 5a. In the channeling direction, less information is recorded by the TOF-SIMS detector since less sputtering occurs by the primary ion beam. Therefore, the intensities of all the elements should be reduced compared to a non-channeling direction. These crystallographic effects in primary grains are seen, for example, in Fig. 5b. In the particular case of the grain in Fig. 5a, Mn, Ni and Co follow the expected crystallographic trends. Lithium, however, shows an increased number of counts. Channeling grains with this high amount of lithium were not found in pristine samples. Therefore, one must conclude that this observation is a result of the electrochemical activity that the cathodes have undergone. It is known that lithium ions may remain trapped at grain boundaries or at triple join points as a result of charge-discharge cycles and contribute to the degradation mechanisms^19^. These trapped ions were identified by Sui *et al*^19^ as “lithium hotspots”, which indicate an incomplete electrochemical reaction inside the NMC grains. In their paper, the presence of these hotspots was also linked with battery degradation and capacity fade. Results in this study show, however, that lithium hotspots are not found at the grain boundaries, but rather in the primary grains. Grain boundaries in Fig. 5 clearly show enhanced intensities, but according to our previous analysis regarding FIB SE imaging of the NMC grains and the analysis of edge effects, these increases in the analytical signal should be attributed to the topography obtained by sputtering at non-normal incidence.

## Conclusion

In this work, a TOF-SIMS detector mounted on a FIB-SEM platform was used to detect lithium in standard NMC cathodes with different state-of-charge, and this technique is proposed as a solution to the detection limitation of EDS for lithium. High-resolution elemental distribution of active elements in NMC cathodes were obtained, allowing the identification of various phenomena resulting from sputtering. Both crystallographic and chemical effects were observed in primary grains in the ion maps. It was shown that edge effects can be correlated with high sample topography in SE images, and these regions on NMC grains were disregarded to avoid false analysis. High intensities of lithium at grain boundaries are the result of edge effects and artifacts in the analysis. Further investigations will be made to automate the identification of edge effects with correlative confocal microscopy. The quantification of lithium content in NMC cathodes with different state of charge was achieved using a calibration curve. This approach is a solution to the existing SIMS quantification method that did not provide meaningful results with the battery materials. At C/10 a polynomial relationship that corresponds to TRIM theoretical simulation of lithium extraction from a NMC matrix was found. An indirect linear relationship was found between lithium content in the NMC compounds (*x*) and measured TOF-SIMS intensities at C/50, which we believe is attributed to SOC heterogeneities within the secondary particles, as well as reconstruction of a surface layer and lithium segregation. Due to the matrix effects that are inherent to the technique, determining the Li concentration with the calibration curve applies only to our standard NMC. A specific experimental calibration curve is needed for each material for which the Li concentration is required because each material will exhibit different emission response to the primary ion-beam sputtering, once again as a result of matrix effects. Work is currently ongoing to quantify the Li content in lithium iron phosphate (LFP) cathodes, but since the primary grains are of the order 100 to 200 nm, there is an enhanced edge effect compared to NMC. Consequently, more work is required to optimize the technique for the analysis of LFP particles.

## Methods

### Cathode sample preparation

A slurry was prepared by mixing NMC-532 powder, conductive carbon and PVdF binder with a net ratio of 93:4:3, and using NMP solvent. The slurry was coated on the aluminum foil and dried at 110 °C under vacuum to remove the solvent and residual moisture. The dry electrode had the loading of 10.5 mg cm^−2^ and the density after calendaring was controlled to 2.9 g cm^−3^.

### Cell assembly and SOC control

The cathode was assembled in a CR2032-type coin cell inside a He-filled glove box, using commercial lithium metal foil (200 µm thick) and ceramic-coated porous polyethylene film (W-Scope) as anode and separator, respectively. The electrolyte was 1 M LiPF_6_ in EC:EMC (3:7). The coin cell was first charged to 4.5 V at 0.1 C and fully discharged to 3.0 V. Then the cells were charged at the desired C-rate (C/10 or C/50) until the calculated capacity to control the degree of lithiation was obtained. We assumed that x = 1 at the fully discharged state and the theoretical capacity is 277.6 mAh g^−1^ where x = 0. The galvanostatic charge and discharge was performed using a cycler (VMP3, BIOLOGICS). For the analysis of cathode electrodes, the coin cells were carefully dismantled in the glove box and the electrodes were washed with DMC and dried before transferring to the instrument used in the chemical and surface analysis.

### Chemical delithiation of NMC powders

NMC-532 powder (5 g) was dispersed in a solution containing acetonitrile (100 ml), and the proper quantities of Br_2_ as oxidizing agent (dibromide, Br_2_, ≥99.99% trace metals basis, Sigma-Aldrich) were added to the solution and stirred magnetically overnight. The final powders were precipitated and rinsed with acetonitrile and ethanol. Before SIMS analysis, the NMC were dried in an oven for 24 hrs.

### TOF-SIMS measurements

Cross-sections of the cycled cathodes were prepared using the Ar ion milling system IM4000PLUS by Hitachi High Technologies to produce a flat surface for the TOF-SIMS analysis and to reduce the occurrence of edge effect at the source. Measurements with a confocal microscope [VK-X series, Keyence Laboratories] revealed a roughness of less than 150 nm at the surface of the milled particles. FIB milling in the FIB-SEM microscope [LYRA3 GT, TESCAN] was also investigated to prepare samples with lower roughness of the particles, but was discarded due to gallium poisoning that limited surfaces for analysis of the prepared trenches and added time/expense for preparation.

TOF-SIMS results were achieved using TESCAN’s Lyra3 GT FIB-SEM microscope. The Cobra Ga^+^ ion column has 2.5 nm resolution at 30 keV, and the SEM is a field emission with a resolution of 2 nm at 5 keV. The microscope is equipped with a TOF-SIMS detector mounted on one of the upper ports of the SEM chamber. This TOF-SIMS analyzer is a result of the collaborative work of TESCAN and TOFWERK AG. The FIB column provides the primary ion source for the TOF-SIMS technique, where the bombardment of the Ga ions on the sample surface, and the resulting collision cascade will eject surface atoms. The ejected atoms, called secondary species, can be neutral, electrons, molecules and ions. The ionized particles (single elements or molecules) produced secondary ions (SI) that were collected in the mass analyzer by applying a potential difference. Upon entry, the SI will be accelerated at 5 kV and drift in the mass analyzer along a fixed distance, called drift length, until they reach the detector that will sort them according to their arrival time. In order for sufficient data to be collected to construct ion maps of the observed region, in this case a raster of 768 × 768 pixels over a region of 15 × 15 μm is conducted with the FIB with 20 nm resolution in both direction (x and y). Data was collected over 100 frames (consisting of complete rasters over the 15 × 15 analysis area). TOF-SIMS provides both ion distributions over the whole analysis area, as well as mass spectra at each location, provided the integration over 100 frames of analysis.

The cathode cross-sections were mounted on a stud and inserted in the FIB-SEM microscope chamber with the cross-section perpendicular to the electron beam. The TOF-SIMS analysis was performed with the sample tilted at a 55° using the TESCAN motorized stage so that the cross-section is perpendicular to the FIB column (which is positioned at a 55° angle relative to the electron column). Since sputtering is a function of the angle of incidence between the incident beam and the sample surface, this step produced good results and facilitated data interpretation. Ga primary ion energy of 30 keV and beam current of 500 pA were used to collect data. A negative potential difference was applied in the TOF-SIMS device in order to collect positive ions, which were the positive-charged elements of interest. The mass-to-charge (m/z) ratios were calibrated using ^7^Li^+^, ^55^Mn^+^ and ^69^Ga^+^.

In order to compute the lithium intensities, multiple regions of interest (ROI) in the analysis regions were defined using the TOFWERK program and the mass spectra relative to those ROI computed. The ROIs were chosen to avoid the porosity in the areas between NMC particle grains, particles located in secondary planes, as well as edges effects as explained in the text. A simple integration of the desired peak was useful to determine the lithium peak intensity.

### XRD measurements

X-ray diffraction (XRD) analyses were performed on the dried cathodes in order to confirm the lithium content in each of the cycled electrodes and the pristine sample. The XRD spectra are collected on a SmartLab diffractometer (Rigaku) with Co Kα radiation. The Rietveld refinement is conducted by using PDXL2 software (Rigaku). The spectra are refined in the R3 ̅m space group. Li and Ni cation mixing is kept at 0.035 (PDF 01-075-3920). Only the lithium content on the Li site is refined. The total Li content in the structure is the sum of the Li on Li site and Li on Ni site.

## Electronic supplementary material

Supplementary Information
