# Supplementary material for: Nanoscale Lithium Quantification in LiXNiyCowMnZO2 as Cathode for Rechargeable Batteries
Source: Sci Rep. 2018 Dec 4;8:17575. doi: 10.1038/s41598-018-33608-3 (PMC6279772; doi:10.1038/s41598-018-33608-3)
Supplement: Supplementary file 1 — Supplementary Information [file 41598_2018_33608_MOESM1_ESM.docx]

**Nanoscale Lithium Quantification in Li_X_Ni_y_Co_w_Mn_Z_O_2_ as Cathode for Rechargeable Batteries.**

**Stéphanie Bessette**1,2**, Andrea Paolella**1**, Chisu Kim**1**, Wen Zhu^1^, P. Hovington**^3^**, Raynald Gauvin^2^ and Karim Zaghib**1,*

1 Hydro-Québec’s Research Institute, Center of Excellence in Transportation Electrification and Energy Storage, Varennes, J0L 1N0, Canada

2 McGill University, Department of Mining and Materials Engineering, Montréal, H3A 0C5, Canada

3. Consulting Hovington, Boucherville, Québec, Canada.

*  [Zaghib.Karim@ireq.ca](mailto:%20Zaghib.Karim@ireq.ca)

+ these authors contributed equally to this work

**Figure S1.** XRD spectra of electrochemically delithiated NMC cathodes.

The (hkl)s of main phase NMC peaks are labelled for a) C/10 and b) C/50 cycling rates. The (018) and (110) peaks are close in the fully lithiated NMC, whereas they are further apart in the partially lithiated structure. In addition, (006) of fully lithiated NMC is seen at ~44^o^, but it is not seen in partially lithiated NMC. Carbon from the binder is seen in some of the spectra as well as aluminum (220) from the electrode collector. The labelled # peak is impurity in the material, seen only at low lithium content.


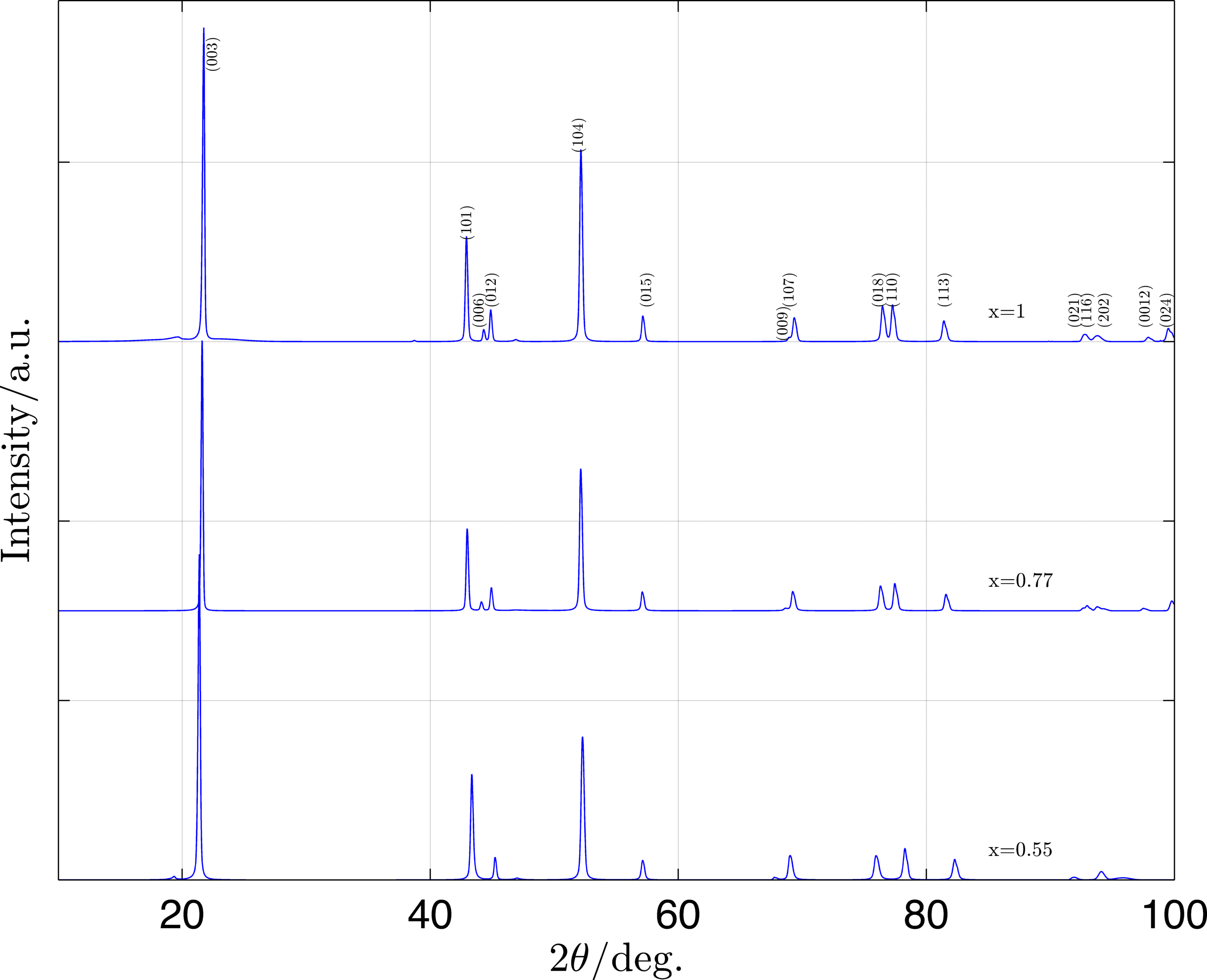


**Figure S2.** XRD spectra of chemically delithiated NMC powder. The (hkl)s of main phase NMC peaks are labelled in the graph. Once again, the (018) and (110) peaks are close in the fully lithiated NMC whereas they are further apart in the partially lithiated structure. In addition, (006) of fully lithiated NMC is at ~44^o^, but it is not seen in partially lithiated NMC.

**Figure S3. a)**TRIM simulations of elemental sputtering yield of lithium atoms in Li_x_[NMC]O_2_ compounds with varying lithium stoichiometry. Simulations were done using *surface sputtering* mode with 100 000 Ga ions with incident energy of 30 keV to reproduce experimental conditions. b) Conversion of experimental intensities (results shown in Figure 3a)) into sputter yields for comparison with theoretical yields. Calculations were made starting from the basic SIMS equation for secondary species signal $I\left( X^{+} \right)=Y_{X}\cdot V\cdot\rho_{X}$ ^35,36^ where $I\left( X^{+} \right)$ is the secondary ion specie of interest, $Y_{X}$is the useful yield, $V$ the analytical volume and the concentration of the desired specie in matrix. The useful yield is by definition the product of the ionization efficiency $\alpha$ of analyte and the transmission efficiency (*T*) of said ions within the SIMS apparatus. A value of 10^-6^ for the useful yield $Y_{X}$was used for calculations, which is typical for FIB-SIMS analysis^36^ . Results show a similar polynomial behavior between the experimental and the simulated sputter yield for lithium ions by gallium primary ions.
